# Quantitative Normative Gait Data in a Large Cohort of Ambulatory Persons with Parkinson’s Disease

**DOI:** 10.1371/journal.pone.0042337

**Published:** 2012-08-03

**Authors:** Chris J. Hass, Paul Malczak, Joe Nocera, Elizabeth L. Stegemöller, Aparna Shukala, Irene Malaty, Charles E. Jacobson, Michael S. Okun, Nick McFarland

**Affiliations:** 1 Center for Movement Disorders and Neurorestoration, University of Florida, Gainesville, Florida, United States of America; 2 Department of Applied Physiology and Kinesiology, University of Florida, Gainesville, Florida, United States of America; 3 Brain Rehabilitation Research Center, Malcom Randall VAMC and Department of Aging and Geriatric Research, University of Florida, Gainesville, Florida, United States of America; 4 Department of Neurology, University of Florida, Gainesville, Florida, United States of America; The Chinese University of Hong Kong, Hong Kong

## Abstract

**Background:**

Gait performance is widely evaluated to assess health status in older adult populations. While several investigators have presented normative values for spatiotemporal gait parameters drawn from older adult populations, the literature has been void of large-scale cohort studies, which are needed in order to provide quantitative, normative gait data in persons with Parkinson’s disease. The aim of this investigation was to provide reference values for clinically important gait characteristics in a large sample of ambulatory persons with Parkinson’s disease to aid both clinicians and researchers in their evaluations and treatments of gait impairment.

**Methodology/Principal Findings:**

Gait performance was collected in 310 individuals with idiopathic Parkinson’s disease as they walked across a pressure sensitive walkway. Fourteen quantitative gait parameters were measured and evaluated with respect to Hoehn and Yahr disease staging and gender. Disease duration and age were controlled for in all analyses. Individuals with the greatest Parkinson’s disability walked significantly slower with shorter steps and stride lengths than the mild and moderately affected groups. Further, the most affected patients spent more time with both feet on the ground, and walked with a wider base of support than the moderately disabled patients. No differences were detected between the mild and moderate disability groups on any of the gait parameters evaluated.

**Conclusions/Significance:**

Reference values for 14 gait parameters in a large cohort of ambulatory patients with Parkinson’s disease are provided and these may be highly useful for assessing and interpreting an individual’s gait dysfunction. It is important for clinicians and researchers to appreciate the lack of change in quantitative parameters as PD patients move from mild to moderate gait impairment.

## Introduction

Gait impairment in Parkinson’s disease (PD) is often compounded by the presence of the other cardinal features of bradykinesia, rigidity, and postural instability. Individuals with PD typically exhibit a slow, short-stepped, shuffling gait with a forward-stooped posture and asymmetrical arm swing. These characteristics alter the metabolic efficiency of gait and lead to performance of routine walking near maximum capacity [1]. For most of us, independent functioning in the community is reliant upon the ability to walk efficiently. Thus, it is not surprising that gait impairment in PD is closely related to poor quality of life and to increased patient distress. Of note, patients themselves have been consistently documented to identify improvement in walking abilities as the most relevant outcome necessary to deem anti-parkinson treatment a success [2].

In order to evaluate treatment effectiveness and progression of gait impairment, a consensus is needed on which gait values are relevant. Although there are many aspects of walking on which the clinician or rehabilitation specialist might focus, gait speed has often been used as measure of status and of potential health care outcome. Indeed, the speed at which individuals walk is relevant to their functioning in the community [3] and is an important predictor of meaningful clinical outcomes such as: length of hospital stay [4] and mortality [5], [6]. Other important spatiotemporal features of gait that are often evaluated clinically include step and stride length, and also step width. These variables provide further insight into the nature of the walking behavior beyond just the simple speed of movement. Moreover, each of these gait features has also been related to other health domains relevant to the older adult including brain atrophy, executive function, memory decline, subtypes of mild cognitive impairment, and fall risk [7]–[12]. Together, these findings suggest that these spatiotemporal variables are not only relevant to defining gait performance, but may also be important indices relating to overall health.

If the movement disorders neurologist and rehabilitation specialist are to make judgments about the normality of a patient’s walking, reference values for clinically and functionally relevant spatiotemporal variables are required for comparison. While several studies have presented normative values of spatiotemporal gait data from healthy young and older adult populations [13]–[19], the literature is void of large-scale cohort studies that provide quantitative, normative gait data in PD. More common in the literature is a comparison of gait performance in a limited sample of persons with PD to a small sample of age-matched healthy participants. Unfortunately, this strategy provides limited clinical utility. Thus, the primary aim of this investigation was to provide reference values for clinically important gait characteristics in a large sample of ambulatory persons with PD at various stages of the disease.

## Results

Demographic and clinical information for the sample is presented in Table 1. The quantitative gait parameters of our large cohort of patients are presented in Table 2. Individuals with the greatest Parkinson’s disability (severely disabled) walked significantly slower with shorter steps and stride lengths than the mild and moderately affected groups (Table 2; both p<0.01). Further, the percent of the gait cycle spent in stance was significantly greater in the more severely disabled group compared to their counterparts (Table 2, both p<0.05). Additionally, patients with moderate disability exhibited a greater cadence (Table 2, p<0.05), and narrower base of support (Table 2, p<0.001) compared to those in the severe disability group. Patients with moderate disability also spent more time in swing (Table 2, p<0.001) and less time in stance (Table 2, p<0.001) and time in double support (Table 2, p<0.001) than those with the highest disease burden. No differences were detected between the mild and moderate parkinsonian disability groups on any of the gait parameters evaluated.

**Table 1:** Patient Demographics.

Age (years)	68±10
Disease Duration (years)	8±6
Sex	Male: 213 (68.7%); Female 97 (31.3%)
Handedness	Right: 90%; Left: 10%
Side of Onset	Right: 49%; Left: 41% Left; Both or unclear: 10%
Hoehn & Yahr	2.2±0.6
UPDRS Motor	25.4±9.9
PDQ Mobility	31.5±27.3
PDQ ADL	28.9±21.5
PDQ Emotional	25.5±22.3
PDQ Stigma	17.3±21.1
PDQ Social	11.8±20.9
PDQ Cognition	27.8±20.8
PDQ Communication	25.8±23.0
PDQ Discomfort	34.1±23.3
Schwab and England (patient)	84.6±11.6
Schwab and England (clinician)	84.0±11.6

Values reported as mean ± one standard deviation.

**Table 2:** Gait Spatiotemporal Variables of Interest (values as reported as mean ±SD).

VARIABLES	FEMALES			MALES			GROUP		
	≤1.5	2–2.5	3–4	≤1.5	2–2.5	3–4	≤1.5	2–2.5	3–4
**Velocity** (m/s)	0.75±0.09	0.92±0.03	0.65±0.04	1.09±0.05	1.02±0.02	0.87±0.04	0.92±0.05*	0.97±0.02*	0.76±0.03
**Cadence** (steps/min)	96±4	105±2	94±2	104±2	103±1	101±2	100±2	104±1*	97±1
**Step time**(s)	0.64±0.19	0.58±0.07	0.65±0.10	0.59±0.11	0.60±0.04	0.82±0.08	0.61±0.11	0.59±0.04*	0.74±0.07
**Step Length** (m)	0.47±0.04	0.53±0.02	0.41±0.02	0.63±0.02	0.59±0.01	0.51±0.02	0.55±0.02*	0.56±0.01*	0.46±0.01
**Stride Length** (m)	0.95±0.08	1.05±0.03	0.83±0.04	1.25±0.04	1.19±0.02	1.04±0.03	1.10±0.05*	1.12±0.02*	1.04±0.03
**Step Width** (m)	0.10±0.01	0.09±0.01	0.12±0.01	0.11±0.08	0.10±0.01	0.12±0.01	0.10±0.01	0.10±0.01*	0.12±0.01
**Swing** (% cycle)	32.0±1.2	34.2±0.4	30.6±0.6	34.6±0.6	34.0±0.2	33.2±0.5	33.3±0.7	34.1±0.2*	31.9±0.4
**Swing time** (s)	0.41±0.05	0.40±0.02	0.40±0.02	0.40±0.03	0.40±0.02	0.45±0.03	0.41±0.03	0.40±0.01	0.42±0.02
**Stance** (% cycle)	68.0±1.2	65.8±0.4	69.4±0.5	65.4±0.6	66±0.2	66.8±0.5	66.7±0.6*	65.9±0.2*	68.1±0.4
**Stance time** (s)	0.87±0.05	0.77±0.02	0.92±0.02	0.76±0.03	0.78±0.01	0.83±0.02	0.81±0.03	0.77±0.01	0.87±0.02
**Single Support** (% cycle)	32.0±2.6	34.1±0.9	30.6±1.3	34.7±1.4	34.1±0.6	35.6±1.0	33.4±1.5	34.1±0.6	33.1±1.0
**Single Support time** (s)	0.41±0.05	0.40±0.02	0.40±0.02	0.40±0.03	0.40±0.01	0.45±0.02	0.41±0.03	0.39±0.01	0.42±0.02
**Double Support** (% cycle)	35.9±5.7	31.8±2.0	39.1±2.8	31.3±3.1	33±1.2	39.8±2.4	33.6±3.3	32.4±1.2*	39.4±1.9
**Double Support time** (s)	0.46±0.25	0.37±0.08	0.52±0.12	0.37±0.13	0.39±0.05	0.69±0.10	0.42±0.14	0.38±0.05*	0.61±0.10

* Statistically different from the severely disabled group (Hoehn and Yahr Stages 3–4). Values reported as mean ± one standard deviation.

A Gender main effect was also identified for numerous variables of interest (p<.05). Male patients walked faster (1.00±.02 m/s versus 0.77±.03) with a faster cadence (103±.1 step/min versus 98±2, p = .015) and produced larger steps (.58±.1 m versus.48±.2, p<0.001) and stride lengths (1.17±.2 versus.94±.3, p<0.001). Further, male patients spent a significantly greater percent of the gait cycle in swing (34±.2 versus 32±.4, p = .01) and less in stance (66±.3 versus 68±.4, p = .01) than their female counterparts.

The component analysis revealed that three factors accounted for 89.3% of the variance in gait behavior. The first factor accounted for 45.0% of the variance in gait performance and was highly influenced by the temporal parameters including step time, swing time, single support time and percentage and double support time and percentage. In accordance with the work of Verghese et al. and Hollman et al. [12], [18], we also termed this factor the rhythm factor. The second factor accounted for 35.8% of the variance loading primarily on velocity, step length, stride length and stance and swing % of the cycle. This factor has previously been coined the ‘pace factor’ [18]. The last factor encompased 8.5% of the variance and loaded highly only on cadence and stance time.

## Discussion

Our study provides normative values for widely used and clinically relevant gait parameters in ambulatory individuals with PD. These norms were derived at cross-section from a large and well characterized cohort of individuals. These comparative norms will help clinicians to compare the gait performance of an individual patient with idiopathic PD to a group of community-residing peers with similar age and gender characteristics. This is the first large population-based study to characterize in detail the relationships between Parkinsonian disability and a range of temporal and spatial gait measures.

Slow gait speed is a significant predictor of poor clinical outcomes and has been associated with increased difficulties in performing activities of daily living, higher risk of falling, hospitalization, lower quality of life, and mortality[13], [20]–[22]. Due to the high relevance of gait speed, there have been several large-scale normative gait studies of gait speed in healthy older adult populations. Bohannon and colleagues [13] in a cohort of “younger” older adults (50–70 years, N = 121), reported comfortable gait speeds ranging on average from 1.35–1.39 m/s. With increasing age, self-selected walking speed is reduced. The mean gait velocity reported in several studies of community dwelling older adults older than 70 years of age range from 1.06–1.22 m/s. For example, Callisaya and colleagues [16] [using similar methods and instrumentation to the present study, reported mean gait speeds averaging 1.16 m/s in men and 1.11 m/s in women from their population-based sample of 411 older adults (average age 72). Recently, in an older cohort of 800 participants (an average age of 80 years), Oh-Park and colleagues [19] documented a slower comfortable walking speed of 0.93 m/s. Of note, most of these studies have purposely excluded individuals with clinical gait abnormalities or diseases affecting gait such as Parkinson’s disease. In the present study, patients with PD walked with an average speed of 0.88 m/s, which is considerably slower than the healthy older adult values reported above. Moreover, as gait impairment in PD becomes clinically more pronounced with advancing disease, the slowest gait velocity is observed in those ambulatory patients with severe disability (Hoehn & Yahr Stages 3–4). Gait speed was on average 24% slower in ambulatory and severely disabled patients (Hoehn & Yahr Stages 3–4) compared to moderately disabled patients (stages <1.5 and 2–2.5). These reductions in gait speed have functional consequences. Forty percent of this cohort of people with Parkinson’s disease do not walk fast enough to cross a street before the light changes (i.e., <.8 m/s), for instance. Further, in older adults survival decreases across the full range of gait speeds, with significant increments per 0.1 m/s [6].

Most studies across a wide range of ages suggest that men exhibit greater gait speed and stride length than women [16], [18], [19], [23]. Our data were consistent with these findings. Gait speed in our cohort of males exceeded that of the females by approximately.22 m/s and stride length in men exceed that in women by approximately.22 m. However, similar to previous findings [18], if gait speed was normalized to stature (in this case leg length), the gender difference was no longer statistically significant. This post-normalization observation supports the notion that differences in gait speed are more influenced by body anthropometrics than by gender.

While gait speed is a well-accepted clinical screen, the magnitudes of other gait parameters also provide important clinical insight. Further, these variables may also be useful in predicting mobility disability and fall risk [11], [24]. For example a 10% decrease from the normal 40% in the swing phase portion of the walking cycle, is associated with greater risk of falls [11]. In the present study of ambulatory patients with PD, the stance phase represented 66.5% of the gait cycle whereas the swing represented 33.5%. However, in the group of severely disabled females the swing phase portion encompased 30% of the gait cycle, perhaps suggesting females with PD are at increased fall risk when compared to their male counterparts. Additionally, in our sample, double limb support occupied 35.1% and single limb support occupied 33.5% of the gait cycle. Comparatively, healthy older adults spend less time in double limb support (27.9% of cycle) and more time in single limb support (36.2% cycle) [18]. Moreover, on average persons in this study with a severe disability, Hoehn & Yahr stages 3–4, spent an additional 1.7% of the gait cycle in stance and 6.4% gait cycle in double support than previously reported in older adults [18], [25]. This increase in stance and double support time represents a decreased efficiency of gait and thus a higher metabolic cost.

Callisaya et al [16] (mean age 72), Hollman et al [18] (ages 70–74, 74–79, 80–85) and Oh-Park et al [19] (mean age 80) provided normative data on cadence, step time, swing time, stance time, stride length, and percent double support using the GAITRite system. Compared to the healthy older adult normative data in the literature, our persons with PD exhibited similar cadence, longer step times, reduced swing time, and greater stance time. Unfortunately, many of the other variables collected herein are mostly absent from the normative literature. Thus, our data will be useful as a reference to guide future investigations.

The results of our factor analysis build upon the findings expressed by Verghese and colleagues [12] and more recently by Hollman and colleagues [18] in community dwelling healthy older adults. Each of these studies identified a “pace factor” typified by velocity and length measures. Pace-related parameters have been studied extensively among older adult populations as many of these variables directly relate to quality of life, fall risk, and even mortality. A second important factor identified in all three studies was a rhythm factor associated most closely with timing. However, parameters within the rhythm domain are also relevant as deleterious alterations in rhythm are related to risk of falling, mobility disability, gray matter atrophy [8] and increased risk of cognitive decline [12].

Several limitations are present and should be taken into account. Participants walked at their natural self-selected speed, not at their maximum possible. Thus, these norms may not relate to the total capacity of the individual. Second, all patients were tested while on anti-Parkinsonian medication. We did not however, standardize where in the medication cycle patients were at the time of testing. All patients self-reported that they were adequately responding to their medicine at the time of testing. This nested cohort study was necessarily restricted to those patients who received a gait assessment from April 2011-October 2011. This was a community based sample but persons were not recruited as a representative population sample. Data was not collected on matched healthy older adults for a direct comparison of the results to a similar community based sample of older adults. Subjects were consecutive patients at a tertiary care movement disorder center, and may or may not parallel the characteristics of a less optimized population. Proportionately, the sample is biased by a large cohort of moderately disabled patients (N = 209) compared to the mild (N = 31) and severe (N = 70). On average 24±.5 steps (12 strides) per participant were collected and analyzed. While there is support that measuring pace and rhythm parameters is highly reliable within this step range, reliability for other parameters, particularly in participants with high step variability, may be improved by evaluating data collected from hundreds of strides.

### Conclusion

This study provides robust norms for quantitative gait parameters in a well-defined cohort of ambulatory community dwelling persons with Parkinson’s disease. These normative gait data are offered to help guide clinicians and scientist in evaluating and interpreting gait impairment.

## Methods

### Participants

Data were collected from three hundred and ten ambulatory patients with a diagnosis of idiopathic PD confirmed by a fellowship trained movement disorders neurologist. Data were collected in the medicated “on” state as part of their routine clinical exam at the Center for Movement Disorders and Neurorestoration in Gainesville, FL from April 2011- October 2011. Patients with deep brain stimulation or other Parkinson-related surgeries or dementia were excluded. The demographics of all participants can be found in Table 1. The study was approved by the University’s Institutional Review Board and all participants provided written informed consent prior to participating.

### Equipment

Gait performance was measured while walking over the GAITRite instrumented walkway system (CIR Systems Inc., Havertown, PA). The 5.8 m×0.9 m pressure sensitive walkway is composed of 18,432 sensors which were activated at foot contact and deactivated at toe-off. The walkway was centered within an isolated 12.2 m×1.37 m collection hallway free from distraction. Data were collected at 120 Hz and processed within the GAITRite Platinum software. We evaluated data from 14 spatial and temporal gait parameters (Table 2).

### Procedures

Participants performed four walks across the GaitRite walkway, beginning and terminating their walks at least 1.5 m before and after the pressure sensitive walkway to minimize acceleration effects. The participants were directed to walk at their natural comfortable pace. A verbal cue was provided to begin walking, though no other cues were provided during the walks nor was any feedback provided regarding their walking performance. Data from all four walks were combined.

A fellowship trained movement disorders neurologist administered part III of the Unified Parkinson’s disease Rating scale and determined the patients modified Hoehn and Yahr (H&Y) Staging. Additionally the Parkinson’s disease questionnaire (PDQ-39) and the Schwab and England (S&E) Disability Scale were completed. Disease duration was determined by subtracting the date of the evaluation from the date of diagnosis with idiopathic PD from the patient’s medical record.

### Data Analysis

The dependent variables of interest (Table 2) were submitted to multivariate analysis of variance (MANOVA), comparing gender and Hoehn and Yahr disability group (Hoehn & Yahr stage ≤1.5, mild disability; Hoehn & Yahr stages 2–2.5, moderate disability; Hoehn & Yahr 3–4, severe disability) as independent variables while controlling for age and disease duration. The significance level for all tests was set at *P*≤.05. We attempted to characterize the gait performance of our participants using principal components factor analysis with varimax rotation. Factors with eigenvalues exceeding 1.0 and parameters with correlation loadings of 0.5 or higher were considered to be significant contributors to the observed behavior. All statistical tests were performed using SPSS 20 (IBM, Armonk, New York).

## References

[1] Katzel LI, Ivey FM, Sorkin JD, Macko RF, Smith B, et al (2012) Impaired economy of gait and decreased six minute walk distance in Parkinson’s disease. Parkinsons Disease 2012: 241754. 10.1155/2012/241754 PMC3171762 21922051

[2] Nisenzon AN, Robinson ME, Bowers D, Banou E, Malaty I, et al (2011) Measurement of patient-centered outcomes in Parkinson’s disease: what do patients really want from their treatment? Parkinsonism Relat Disord 17: 89–94. 20952243 10.1016/j.parkreldis.2010.09.005 PMC3034811

[3] Robinett CS, Vondran MA (1988) Functional ambulation velocity and distance requirements in rural and urban communities. Phys Ther 68: 1371–1372. 3420171 10.1093/ptj/68.9.1371

[4] Rabadi MH, Blau A (2005) Admission ambulation velocity predicts length of stay and discharge disposition following stroke in an acute rehabilitation hospital. Neurorehabil Neural Repair 19: 20–26. 15673840 10.1177/1545968304272762

[5] Ostir GV, Kuo YF, Berges IM, Markides KS, Ottenbacher KJ (2007) Measures of lower body function and risk of mortality over 7 years of follow-up. Am J Epidemiol 166: 599–605. 17566063 10.1093/aje/kwm121

[6] Studenski S, Perera S, Patel K, Rosano C, Faulkner K, et al (2011) Gait speed and survival in older adults. JAMA 305: 50–58. 21205966 10.1001/jama.2010.1923 PMC3080184

[7] Nordin E, Moe-Nilssen R, Ramnemark A, Lundin-Olsson L (2010) Changes in step-width during dual-task walking predicts falls. Gait Posture 32: 92–97. 20399100 10.1016/j.gaitpost.2010.03.012

[8] Rosano C, Aizenstein H, Brach J, Longenberger A, Studenski S, et al (2008) Special article: gait measures indicate underlying focal gray matter atrophy in the brain of older adults. J Gerontol A Biol Sci Med Sci 63: 1380–1388. 19126852 10.1093/gerona/63.12.1380 PMC2648808

[9] Rosano C, Brach J, Longstreth WT Jr, Newman AB (2006) Quantitative measures of gait characteristics indicate prevalence of underlying subclinical structural brain abnormalities in high-functioning older adults. Neuroepidemiology 26: 52–60. 16254454 10.1159/000089240

[10] Rosano C, Studenski SA, Aizenstein HJ, Boudreau RM, Longstreth WT Jr, et al (2012) Slower gait, slower information processing and smaller prefrontal area in older adults. Age Ageing 41: 58–64. 21965414 10.1093/ageing/afr113 PMC3234076

[11] Verghese J, Holtzer R, Lipton RB, Wang C (2009) Quantitative gait markers and incident fall risk in older adults. J Gerontol A Biol Sci Med Sci 64: 896–901. 19349593 10.1093/gerona/glp033 PMC2709543

[12] Verghese J, Wang C, Lipton RB, Holtzer R, Xue X (2007) Quantitative gait dysfunction and risk of cognitive decline and dementia. J Neurol Neurosurg Psychiatry 78: 929–935. 17237140 10.1136/jnnp.2006.106914 PMC1995159

[13] Bohannon RW, Andrews AW, Thomas MW (1996) Walking speed: reference values and correlates for older adults. J Orthop Sports Phys Ther 24: 86–90. 8832471 10.2519/jospt.1996.24.2.86

[14] Bohannon RW, Williams Andrews A (2011) Normal walking speed: a descriptive meta-analysis. Physiotherapy 97: 182–189. 21820535 10.1016/j.physio.2010.12.004

[15] Callisaya ML, Blizzard L, Schmidt MD, Martin KL, McGinley JL, et al (2011) Gait, gait variability and the risk of multiple incident falls in older people: a population-based study. Age Ageing 40: 481–487. 21628390 10.1093/ageing/afr055

[16] Callisaya ML, Blizzard L, Schmidt MD, McGinley JL, Srikanth VK (2010) Ageing and gait variability–a population-based study of older people. Age Ageing 39: 191–197. 20083617 10.1093/ageing/afp250

[17] Hinman JE, Cunningham DA, Recjnitzer PA, Paterson DH (1988) Age-related changes in gait speed of walking. Med Sci Sports Exerc 20: 161–166. 3367751 10.1249/00005768-198820020-00010

[18] Hollman JH, McDade EM, Petersen RC (2011) Normative spatiotemporal gait parameters in older adults. Gait Posture 34: 111–118. 21531139 10.1016/j.gaitpost.2011.03.024 PMC3104090

[19] Oh-Park M, Holtzer R, Xue X, Verghese J (2010) Conventional and robust quantitative gait norms in community-dwelling older adults. J Am Geriatr Soc 58: 1512–1518. 20646103 10.1111/j.1532-5415.2010.02962.x PMC2955162

[20] Abellan van Kan G, Rolland Y, Andrieu S, Bauer J, Beauchet O, et al (2009) Gait speed at usual pace as a predictor of adverse outcomes in community-dwelling older people an International Academy on Nutrition and Aging (IANA) Task Force. J Nutr Health Aging 13: 881–889. 19924348 10.1007/s12603-009-0246-z PMC12878092

[21] Frank JS, Patla AE (2003) Balance and mobility challenges in older adults: implications for preserving community mobility. Am J Prev Med 25: 157–163. 14552940 10.1016/s0749-3797(03)00179-x

[22] Judge JO, Schechtman K, Cress E (1996) The relationship between physical performance measures and independence in instrumental activities of daily living. The FICSIT Group. Frailty and Injury: Cooperative Studies of Intervention Trials. J Am Geriatr Soc 44: 1332–1341. 8909349 10.1111/j.1532-5415.1996.tb01404.x

[23] Samson MM, Crowe A, de Vreede PL, Dessens JA, Duursma SA, et al (2001) Differences in gait parameters at a preferred walking speed in healthy subjects due to age, height and body weight. Aging (Milano) 13: 16–21. 11292147 10.1007/BF03351489

[24] Brach JS, Studenski SA, Perera S, VanSwearingen JM, Newman AB (2007) Gait variability and the risk of incident mobility disability in community-dwelling older adults. J Gerontol A Biol Sci Med Sci 62: 983–988. 17895436 10.1093/gerona/62.9.983 PMC2858390

[25] Murray MP, Kory RC, Clarkson BH (1969) Walking patterns in healthy old men. J Gerontol 24: 169–178. 5789252 10.1093/geronj/24.2.169

