# Correction: Quantitative Normative Gait Data in a Large Cohort of Ambulatory Persons with Parkinson’s Disease

**DOI:** 10.1371/annotation/d4b5158e-0dd1-4e14-b03a-1af4d5f06c0e

**Published:** 2012-10-19

**Authors:** Chris J. Hass, Paul Malczak, Joe Nocera, Elizabeth L. Stegemöller, Aparna Shukala, Irene Malaty, Charles E. Jacobson, Michael S. Okun, Nick McFarland

The fifth author's name is spelled incorrectly. The correct name is: Aparna Wagle Shukla.

The correct citation is: Hass CJ, Malczak P, Nocera J, Stegemöller EL, Wagle Shukla A, et al. (2012) Quantitative Normative Gait Data in a Large Cohort of Ambulatory Persons with Parkinson’s Disease. PLoS ONE 7(8): e42337. doi:10.1371/journal.pone.0042337

